# The Zwitterionic Cell Wall Teichoic Acid of *Staphylococcus aureus* Provokes Skin Abscesses in Mice by a Novel CD4+ T-Cell-Dependent Mechanism

**DOI:** 10.1371/journal.pone.0013227

**Published:** 2010-10-07

**Authors:** Christopher Weidenmaier, Rachel M. McLoughlin, Jean C. Lee

**Affiliations:** Channing Laboratory, Department of Medicine, Brigham and Women's Hospital and Harvard Medical School, Boston, Massachusetts, United States of America; National Institutes of Health, United States of America

## Abstract

Zwitterionic polysaccharide (ZPS) components of the bacterial cell envelope have been shown to exert a major histocompatibility complex (MHC) II-dependent activation of CD4+ T cells, which in turn can modulate the outcome and progression of infections in animal models. We investigated the impact of zwitterionic cell wall teichoic acid (WTA) produced by *Staphylococcus aureus* on the development of skin abscesses in a mouse model. We also compared the relative biological activities of WTA and capsular polysaccharide (CP), important *S. aureus* pathogenicity factors, in abscess formation. Expression of both WTA and CP markedly affected the ability of *S. aureus* to induce skin abscess formation in mice. Purified wild-type zwitterionic WTA was more active in inducing abscess formation than negatively charged mutant WTA or purified CP8. To assess the ability of purified native WTA to stimulate T cell proliferation in vitro, we co-cultivated WTA with human T-cells and antigen presenting cells in the presence and absence of various inhibitors of MHC-II presentation. Wild-type WTA induced T cell proliferation to a significantly greater extent than negatively charged WTA. T cell activation was dependent on the presentation of WTA on MHC II, since inhibitors of MHC II-dependent presentation and antibodies to MHC II significantly reduced T cell proliferation. T cells activated in vitro with wild-type WTA, but not negatively charged WTA, induced abscess formation when injected subcutaneously into wild-type mice. CD4−/− mice similarly injected with WTA failed to develop abscesses. Our results demonstrate that the zwitterionic WTA of *S. aureus* induces CD4+ T-cell proliferation in an MHCII-dependent manner, which in turn modulates abscess formation in a mouse skin infection model. An understanding of this novel T cell-dependent host response to staphylococcal abscess formation may lead to the development of new strategies to combat *S. aureus* skin and soft tissue infections.

## Introduction

**Figure 1:**
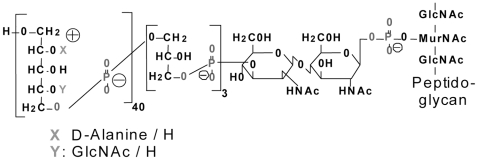
*Staphylococcus aureus* WTA is a zwitterionic cell wall polymer composed of ∼40 ribitol phosphate repeating units modified with *N*-acetylglucosamine and D-alanine. The negatively charged phosphodiester bonds and the positively charged D-alanine residues are responsible for the zwitterionic nature of the polymer.

Wall teichoic acid (WTA) of *Staphylococcus aureus* is a zwitterionic cell wall glycopolymer composed of ∼40 ribitol phosphate repeating units modified with *N*-acetylglucosamine and D-alanine [1], [2]. The negatively charged phosphate groups in the ribitol repeating unit and the ester-linked D-alanine residues contribute to the zwitterionic charge of WTA (Fig. 1). The presence of WTA-like molecules in most Gram-positive bacteria prompted scientists to focus on the role of WTA in cell wall function [3], [4] and turn-over [5]. More recently, defined mutants of *S. aureus* that lack WTA or have altered WTA structures have facilitated experiments to elucidate in greater detail the role of WTA in staphylococcal pathogenesis [6]–[10]. WTA has been implicated in the adhesion of *S. aureus* to human epithelial and endothelial cells [9]–[11], and expression of WTA has been shown to be essential for *S. aureus* nasal colonization of cotton rats [9], [11]. A *S. aureus* mutant lacking WTA showed attenuated virulence in a rabbit model of endocarditis [10]. Moreover, purified WTA was able to induce intraabdominal abscesses when rats were inoculated by the intraperitoneal route [12]. *S. aureus* mutants in the *dlt* operon, which mediates D-alanylation of WTA, exhibit a negatively charged cell surface and are more sensitive to cationic antimicrobial peptides than the parental strain [13], [14].

WTA is expressed constitutively [1], whereas *S. aureus* capsular polysaccharides (CPs) are expressed in a tightly regulated manner [15], [16]. Most clinical isolates of *S. aureus* express either capsule type 5 (CP5) or 8 (CP8). However, 20–25% of clinical isolates produce no capsule due to a variety of mutations within the conserved capsule biosynthesis operon [17], [18].

WTA exhibits a zwitterionic charge, and advances in the field of glyco-immunology have led to the finding that zwitterionic polysaccharides (ZPS) exert a direct activity on the adaptive immune system, thereby modulating the development of bacterial infections [19]. ZPS, such as complex CPs produced by specific bacterial pathogens, directly elicit immune responses by activating various T cells subtypes in the absence of protein carriers [20], [21]. ZPS activated T cells modulate the outcome of infection or colonization by different bacterial species [19], [22]. CP5 and CP8 produced by *S. aureus* enhance staphylococcal virulence [23]–[25], and purified zwitterionic CP8 induces intraabdominal abscesses in rats in a CD4 T cell dependent manner [12]. In a wound infection model, McLoughlin et al. demonstrated that the presence of CD4 T cells modulated CXC chemokine production at the *S. aureus* infection site in an IFN-γ dependent manner, which led to a massive recruitment of neutrophils, and this effect was mediated by CP8 [26]. These findings suggest that staphylococcal ZPS are important factors for T cell dependent immune stimulation, which strongly influences the outcome of infections.

Skin and soft tissue infections are the most common types of *S. aureus* infections and occur in the hospital as well as in the community, affecting hosts without predisposing risk factors [27], [28]. Since bacterial ZPS are associated with abscess formation [12], [29], we explored the contribution of zwitterionic WTA and CPs in a mouse skin infection model. We demonstrate here that WTA activates T cells in a MHC II dependent manner, and that T cells activated in vitro by WTA induce skin abscesses in mice. These novel findings link staphylococcal skin infections to recent discoveries of glyco-immunology and may lead to novel approaches to combat skin infections by *S. aureus.*

## Results

### WTA and Capsule Impact Abscess Formation When Live Bacteria Are Injected Subcutaneously into the Flanks of Mice

We used a mouse model to study the ability of various *S. aureus* strains to induce skin infections that closely resemble staphylococcal skin infections in humans. *S. aureus* cells mixed with cytodex beads were injected subcutaneously into the flanks of mice. The cytodex beads allow for formation of a localized abscess even at low inocula or with low concentrations of purified WTA.

To assess the relative roles played by *S. aureus* WTA and CPs in skin infections, we transduced the *tagO* mutation into the CP5+ strain Newman and its isogenic acapsular *cap5O* mutant. The resulting *tagO* deletion mutants lacked WTA in their cell wall as evidenced by the low phosphorus content of cell wall fractions, but their doubling times were similar to that of the parental strains under in vitro conditions (Fig. S1). The Newman *tagO* mutant was complemented by introducing a wild-type copy of *tagO* on plasmid prB*tagO*. In addition, we used previously described mutants of the acapsular SA113 strain that either lack WTA (*tagO*) [9] or the D-alanine esters (*dltA*) [13] in the repeating WTA subunits. The *dltA* mutation was transduced into Newman, and the phenotype of the resulting mutant was consistent with the absence of positive charges in the teichoic acids (Fig. S1).

**Figure 2:**
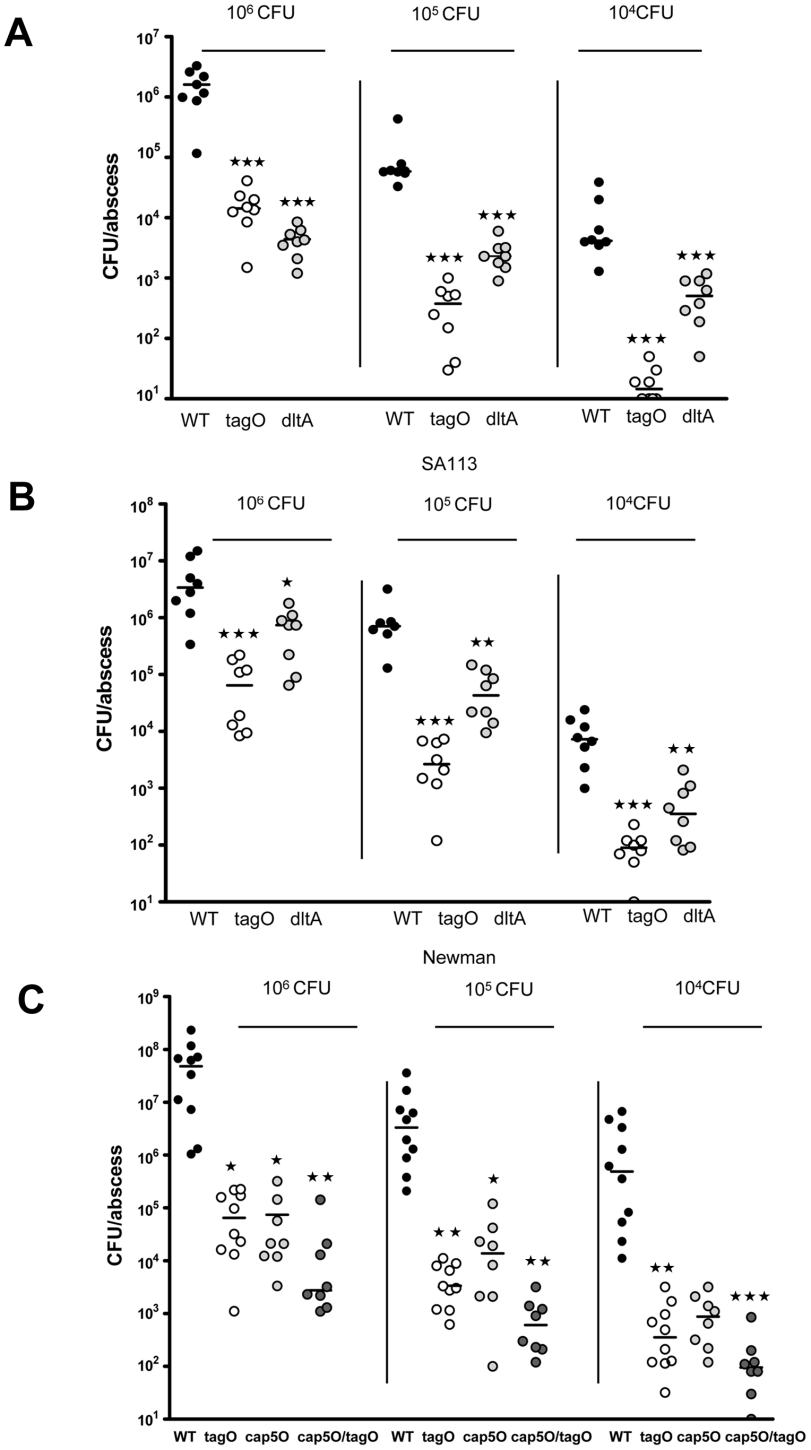
Strains with no WTA or structurally altered WTA are deficient in induction of s.c. abscesses. 10^6^ to 10^4^ CFU *S. aureus* was injected s.c into the flanks of mice. After 48 h, the mice were euthanized, and each abscess was excised, homogenized, and cultured quantitatively. Horizontal bars represent group median values, n = 8. A) The Sa113*dlt*A mutant (negatively charged teichoic acids) and the *tag*O mutant (no WTA) were attenuated compared to the wt (all *p*-values are 0.0002). B) Newman *tagO* (*p* = 0.0002 at 10^6^; *p* = 0.0003 at 10^5^, *p* = 0.0002 at 10^4^) and the *dltA* mutant (*p* = 0.0047 at 10^6^; *p* = 0.0006 at 10^5^, *p* = 0.007 at 10^4^) were significantly less virulent in the abscess model compared to the wt, n = 8. All *p*-values were determined by Mann-Whitney analysis. C) Newman wt, *tagO*, isogenic acapsular mutant *cap5O* and the *cap5O/tagO* double mutant were grown on CSA plates to enhance CP production. The Newman *tagO* (*p*<0.05 at 10^6^; *p*<0.01 at 10^5^, *p*<0.01 at 10^4^) and *cap5O* (*p*<0.01 at 10^6^; *p*<0.05 at 10^5^, *p*>0.05 at 10^4^) mutants and the double mutant (*p*<0.001 at 10^6^; *p*<0.001 at 10^5^, *p*<0.001 at 10^4^) were significantly inhibited in abscess formation compared to the wt. The mutants showed no significant difference when compared to each other. P-values for Figure 2C were determined by Kruskal-Wallis one-way ANOVA with Dunn's multiple comparison test. **★** *p*<0.05*;* **★★** *p*<0.01, **★★★** *p*<0.001.

*S. aureus* wild-type strains SA113 and Newman at inocula ranging from 10^6^ to 10^4^ cfu were potent inducers of subcutaneous abscesses in mice (Fig. 2). The *tagO* mutants of both bacterial strains were significantly attenuated in their ability to induce abscesses, whereas the *dltA* mutants showed an intermediate phenotype at the lower inocula (Figs. 2A and 2B and Supplemental Fig. 2). Consistent with previous data with a CP8+ strain [22], the acapsular Newman *cap5O* mutant was also attenuated for subcutaneous abscess formation (Fig. 2C). When the bacteria were grown on Columbia salt agar to enhance capsule expression (Fig. 2C), strain Newman provoked a higher bacterial burden per abscess than when it was grown on tryptic soy agar (Fig. 2B). The Newman *tagO* and *cap5O* mutants were both significantly attenuated for abscess induction when compared to the parental strain. A double mutant of Newman that expresses neither capsule nor WTA was more attenuated for abscess formation than the single mutants (Fig. 2C), but the differences did not reach statistical significance. These data suggest that both WTA and CP promote *S. aureus* skin abscess formation when live bacteria are used as the inocula.

### Purified WTA Induces Skin Abscesses More Efficiently than Purified CP8

**Figure 3:**
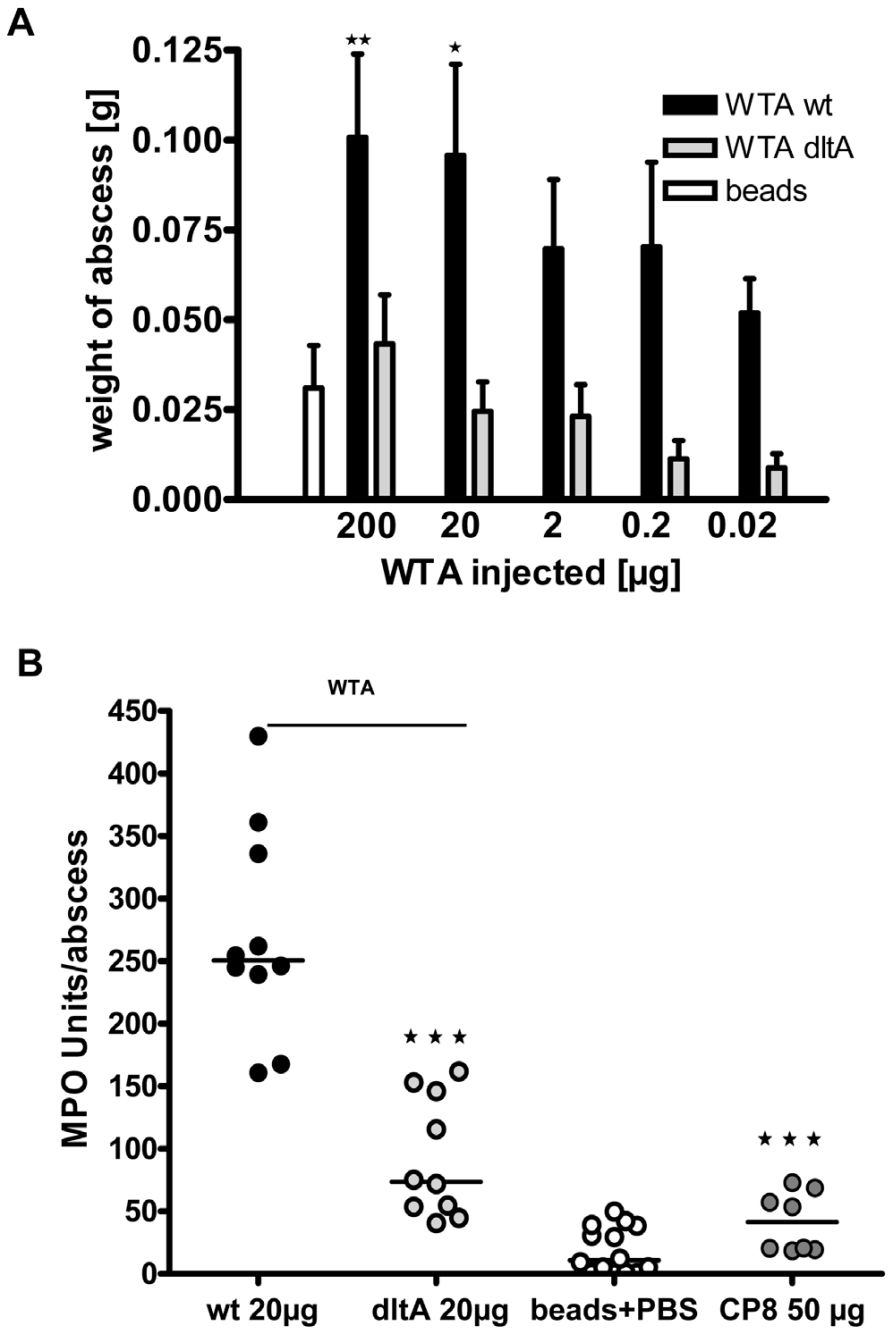
Purified WTA is a potent abscess inducer. WTA was purified from wt SA113 or the *dltA* mutant, mixed with cytodex beads, and injected s.c. into mouse flanks. A) The weights of the abscesses were determined after 48 h and expressed as mean ± SEM, n = 8. The zwitterionic WTA purified from SA113 induced well-defined abscesses, whereas the negatively charged *dltA* WTA was only a weak abscess inducer (wt WTA compared to beads *p* = 0.0062 at 200 µg; *p* = 0.0381 at 20 µg. *dltA* WTA compared to beads yielded p values >0.05 at all doses. All *p*-values were determined by two-tailed Student t-tests). B) 20 µg WTA purified from wild-type SA113 or the *dltA* mutant was mixed with cytodex beads and injected into the flanks of mice. After 48 h, the abscesses were excised, homogenized, and MPO activity was determined to assess the intensity of the host response. Wt WTA induced strong MPO activity, indicative of neutrophil recruitment, whereas *dltA* WTA and CP8 were considerably less active (*p*-values <0.0001 by Mann-Whitney analyses). **★** *p*<0.05*;* **★★** *p*<0.01, **★★★** *p*<0.001.

To further assess its biological activity, we purified WTA from the acapsular strains SA113 wild-type (zwitterionic WTA) and SA113 *dltA* (anionic WTA). WTA purity was assessed by ^1^H NMR, phosphate content, the absence of nucleic acids (<0.1%), protein (<0.1%), and endotoxin (<1 EU/mg). Sterile abscesses were induced by injection of sterile cytodex beads mixed with purified wt WTA at doses ranging from 200 to 0.02 µg. After 48 h, abscesses were excised and quantified by their weight. Purified wt WTA was a potent inducer of abscess formation at doses as low as 0.02 µg (Fig. 3A). WTA purified from the *dltA* mutant or beads alone showed minimal activity under these conditions (Fig. 3A). Administration of *dltA* WTA resulted in the recruitment of fewer neutrophils in the abscessed tissue than wt WTA (Fig. S2). We quantified the host response by measuring neutrophil accumulation at the infection site with the myeloperoxidase (MPO) assay (Fig. 3B) [30]. MPO is present in the azurophilic granules of neutrophils and is routinely used to assess the tissue inflammatory responses [31] and abscess formation. MPO activity in abscesses provoked by 20 µg wt WTA after 48 h was significantly (P<0.0001) higher than that induced by 20 µg *dltA* WTA. Despite its potent activity in the induction of intraabdominal abscesses [12], purified CP8 exhibited only minimal potency in the s.c. abscess model, even at a 50 µg dose.

### Purified WTA Fails to Induce Neutrophil Chemotaxis or Proinflammatory Cytokines

**Figure 4:**
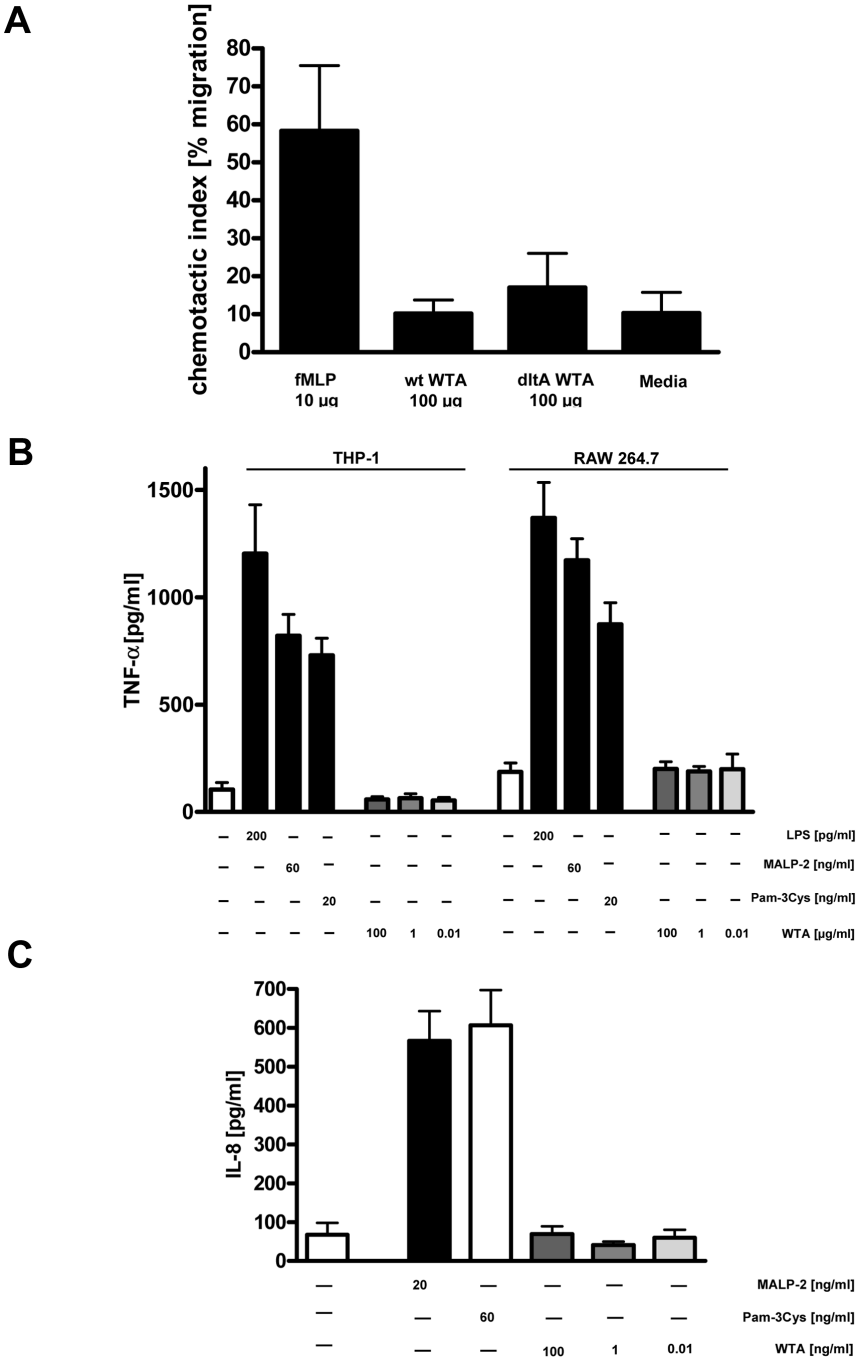
WTA fails to directly induce chemotactic migration or cytokine induction. A) The chemotactic activity of WTA was assessed in a transwell assay. Shown are means (n = 3) and SD of the chemotactic index (% of cells that migrated from the upper compartment towards the stimulus in the lower compartment). The positive control fMLP induced strong chemotactic migration whereas wt and *dltA* WTA failed to affect neutrophil migration (p>0.05 for WTA vs. media by Mann-Whitney analyses). B) Production of TNF-α in the human monocyte cell line THP-1 and the mouse macrophage cell line RAW 264.7 after stimulation for 8 h. LPS, Pam3Cys and MALP-2 stimulated a potent response whereas wt WTA at 100, 1 or 0.01 µg/ml failed to induce cytokine production (WTA vs. media >0.05 by two-tailed Student t-test). Shown are means and SD, n = 3. C) Production of IL-8 in TLR-2 transfected HEK293 cells after stimulation for 10 h. Pam3Cys, MALP-2 stimulated a potent response whereas wt WTA failed to induce cytokine production at concentrations of 100,1 or 0.01 µg/ml. Shown are means and SD, n = 3 (WTA vs. media *p*>0.05 by two-tailed Student t-test ).

To investigate whether purified WTA exhibited direct chemotactic activity, we assayed chemotaxis of human neutrophils in response to WTA in a transwell assay. WTA purified from *S. aureus* SA113 or the SA113 *dltA* mutant failed to induce significant chemotaxis compared to the authentic chemoattractant fMLP (Fig. 4A).

Activation of pro-inflammatory responses via pattern recognition receptors like Toll-like receptors (TLRs) could also lead to pronounced inflammation and modulate abscess formation. Therefore, we tested whether purified WTA stimulates pro-inflammatory cytokines in macrophages or in HEK293 cells transfected with TLR-2. These experiments not only interrogated WTA proinflammatory activity but also enabled us to exclude functional contaminants in our purified WTA preparation. We measured production of TNF-α by human monocytes (THP-1 cells) or mouse macrophages (RAW 264.7 cells) after stimulation with WTA or with the TLR agonists LPS, MALP-2, or Pam3Cys as positive controls. We stimulated HEK293 cells transfected with TLR-2 with WTA, MALP-2, or Pam3Cys, and assayed for IL-8 production in the culture supernatants. Purified WTA at concentrations as high as 100 µg/ml showed no proinflammatory activity in THP-1 or RAW 264.7 cells (Fig. 4B) or in TLR-2 expressing HEK293 cells (Fig. 4C). This is consistent with previous reports that demonstrated the inability of WTA to directly activate host cells in a manner typical of classical pathogen associated molecular pattern molecules [32].

### WTA activates proliferation of human T cells in a MHC II dependent manner

**Figure 5:**
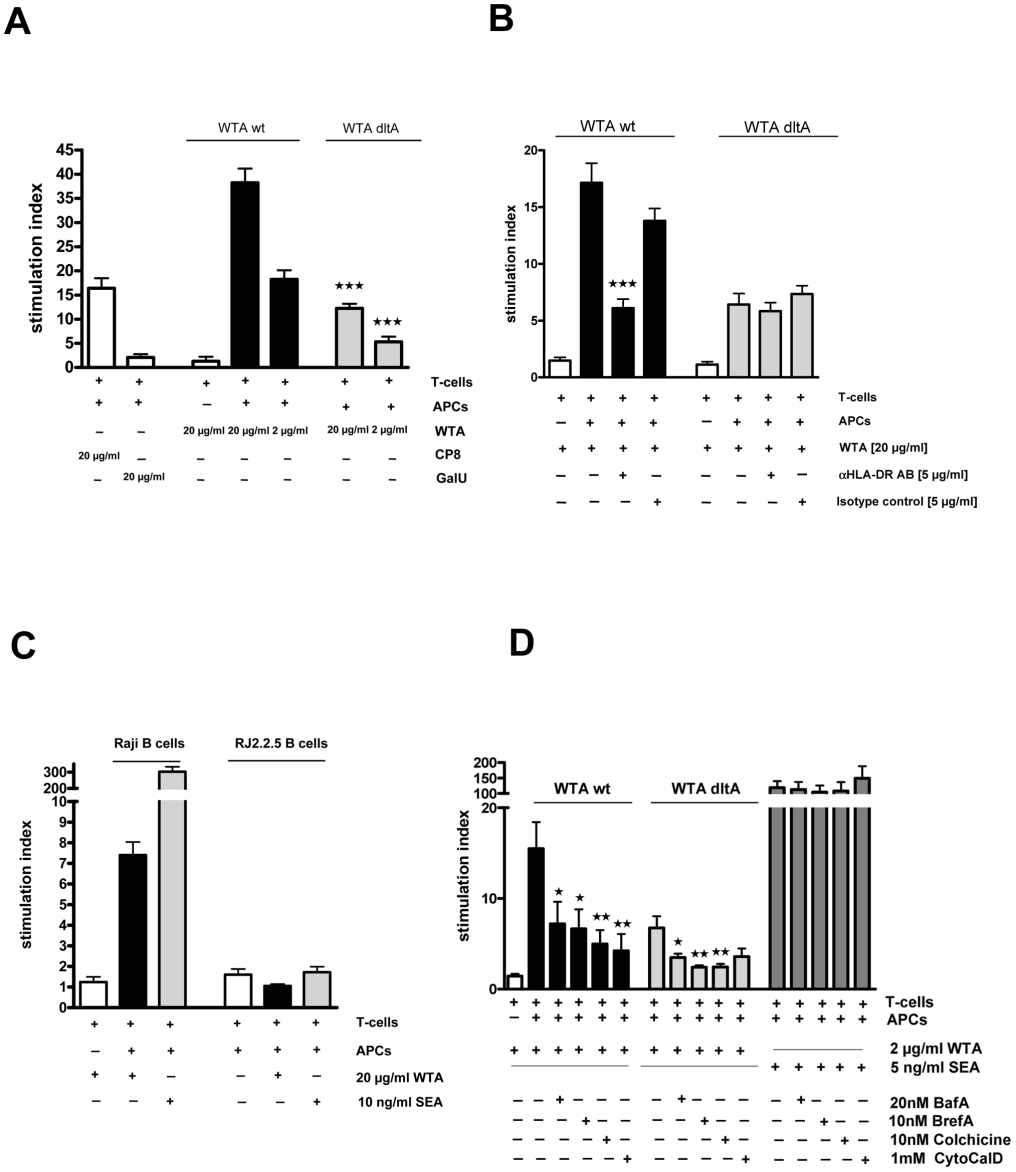
WTA stimulates in vitro T-cell activation by a MHC-II dependent mechanism. T cells and APCs were incubated with different concentrations of WTA purified from SA113 wt or the *dltA* mutant. The stimulation index was calculated by normalizing on wells with APCs and T-cells but no stimulating antigen. A) wt WTA stimulated T-cell proliferation in a dose-dependent manner, whereas *dltA* WTA and CP8 were considerably less active (*p*<0.0001 for wt WTA vs. *dltA* WTA at 20 and 2 µg, *p* = 0.0003 for wt WTA vs. CP8). The negatively charged GalU showed no activity. SEA was considerably more active than WTA (panel D). Values represent means ± SEM, n = 6. B) T-cell proliferation assays with WTA from wt and *dltA* were performed in the presence of blocking Abs to the MHC class II molecules and the appropriate isotype control. An antibody to HLA-DR (5 µg/ml) inhibited T-cell proliferation induced by zwitterionic wt WTA (*p*<0.0001) but not negatively charged *dltA* WTA. Shown are means ± SEM. n = 5. C). WTA induced T-cell proliferation only with Raji cells as APCs but not when APCs were from the MHC II negative cell line RJ2.5.5. Shown are means and SEM, n = 4. D) T cells were incubated with different inhibitors of the MHC II antigen processing pathway. The inhibitors bafilomycin A (BafA), brefeldin A (BrefA), colchicine, and cytochalasin D (CytoCalD) decreased T-cell proliferation induced by wt WTA (wt WTA with BafA: *p* = 0.0399; wt WTA with BrefA: *p* = 0.0230; wt WTA with Colchicine: *p* = 0.0042; wt WTA with CytoCalD; *p* = 0.0036) and *dltA* WTA (*dlt* WTA with BafA: *p* = 0.0220; *dlt* WTA with BrefA: *p* = 0.0026; *dlt* WTA with Colchicine: *p* = 0.0032; *dlt* WTA with CytoCalD: *p* = 0.0527). Shown are means ± SEM, n = 4. All *p*-values were determined by the two-tailed Student t-test. **★** *p*<0.05*;* **★★** *p*<0.01, **★★★** *p*<0.001.

The induction of intraperitoneal abscesses by other zwitterionic polysaccharides is based on their ability to directly activate T-cells, which in turn modulate cellular responses at the infection site [14], [28]. Therefore, we cultured nylon wool-purified human T-cells or column-purified CD4+ T cells with irradiated antigen-presenting cells (APCs) and wt or *dltA* WTA. *S. aureus* enterotoxin A (SEA) and polygalacturonic acid (GalU) were used as positive and negative controls, respectively. Wt WTA elicited a potent dose-dependent proliferative response in human T cells on day 6 (Fig. 5A). The T-cell response to wt WTA was significantly higher than that exhibited by CP8, *dltA* WTA, or beads alone (Fig. 5A). The negatively charged polygalacturonic acid failed to induce T-cell proliferation, and the *S. aureus* superantigen SEA induced a significantly higher T-cell response than proliferation induced by either of the WTA antigens (Fig. 5D).

To determine whether T-cell proliferation induced by purified WTA was mediated by MHC class I or II molecules, we performed assays in the presence of blocking antibodies (Abs) and isotype control Abs. Wt WTA induced T-cell proliferation was dependent on the MHC class II molecule HLA-DR since T cell proliferation was reduced significantly in the presence of blocking Abs to HLA-DR (Fig. 5B), but not with Abs to HLA-DP, HLA-DQ, or HLA-A, -B, or –C (data not shown). The residual activity of the *dltA* WTA was not inhibited by any of the Abs. In assays utilizing the human Burkitt lymphoma cell line (Raji) and its MHC class II transcriptional mutant cell line (RJ2.2.5), WTA stimulated T-cell proliferation only when the MHC-II–expressing Raji cells, but not the RJ2.2.5 cells, were incubated with T-cells and WTA (Fig. 5C). Inhibitors of the intracellular antigen-processing pathways that are required for MHC II-dependent antigen presentation were used at concentrations that had no effect on the stimulatory activity of the *S. aureus* superantigen SEA (Fig. 5D). The inhibitors Bafilomycin A (BafA), Brefeldin A (BrefA), Colchicine, and Cytochalasin D (CytoCalD) reduced the T-cell proliferation induced by wt WTA and *dltA* WTA, providing evidence that, unlike superantigens, WTA requires intracellular processing for efficient MHC II presentation to T cells.

### WTA Stimulated Mouse T Cells Modulate Abscess Formation in the S.C Abscess Model

**Figure 6:**
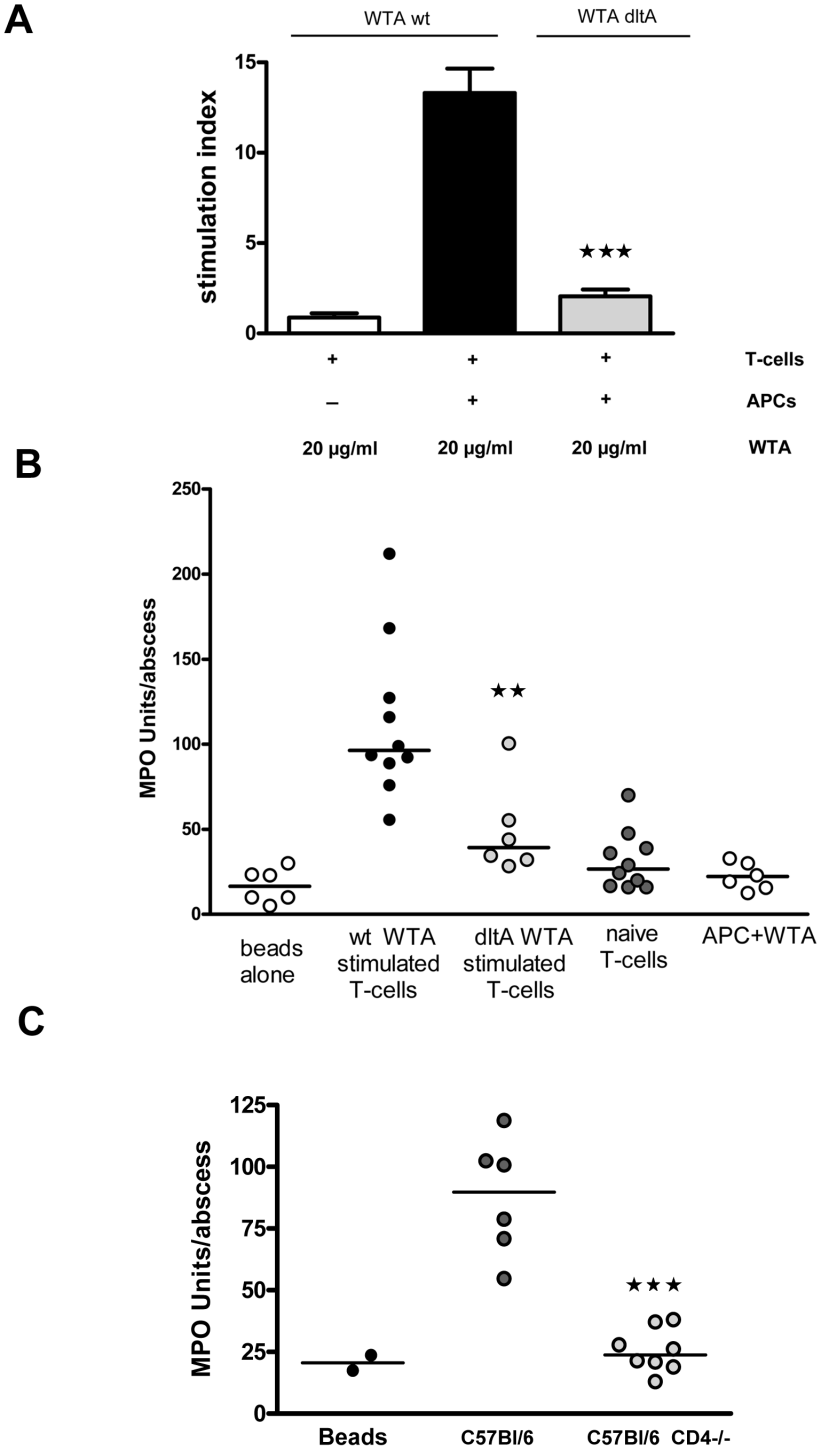
WTA-stimulated mouse T cells modulate abscess formation. A) T cells and APCs were incubated with 20 µg WTA purified from SA113 wt or the *dltA* mutant. The stimulation index was calculated by normalizing on wells with APCs and T-cells but no stimulating antigen. wt WTA stimulated T-cell proliferation more efficiently than *dltA* WTA (*p*<0.0001 for wt WTA vs. *dltA* WTA at 20 µg). Values represent means ± SEM, n = 3. B) Mouse T cells were incubated with APCs in the presence of WTA purified from wild-type SA113 or the *dltA* mutant. Additional T cells were incubated with APCs and no stimulus. After 6 days CD4+ T cells were purified, and 3×10^5^ cells were mixed with cytodex beads and injected s.c. into mice. The abscesses were excised after 48 h, and MPO activity was measured in the abscess homogenate. T cells stimulated with wt WTA showed a significantly greater ability to provoke abscess formation than T cells stimulated with *dltA* WTA (*P* = 0.0075 by Mann Whitney analysis). Shown are the values for individual mouse samples, and horizontal bars represent the medians. C) 20 µg of purified WTA or PBS was mixed with cytodex beads and injected into the flanks of C57BL/6 mice or isogenic mice with no functional CD4+ T cells. Shown are the values for individual mouse abscesses, and horizontal bars represent the medians. Abscess MPO activity was higher in WT vs. CD4 −/− mice (*P* = 0.0007) by Mann Whitney analysis. **★** *p*<0.05*;* **★★** *p*<0.01, **★★★** *p*<0.001.

The role of T-cell activation in the modulation of WTA-induced abscess formation was assessed by T-cell transfer experiments. *S. aureus* wt WTA stimulated a potent proliferative response in mouse T cells after 6 days, whereas *dltA* WTA was less active (Fig. 6A). In T cell transfer assays, purified mouse CD4+ T cells were cultured in vitro for 6 days with WTA and irradiated APCs. The stimulated T cells were then purified, assayed for purity by FACS analysis, mixed with cytodex beads, and injected s.c. into mouse flanks. Abscess formation was quantified by MPO activity in the abscessed tissue after 48 h. In dose-dependency experiments, we found that 3×10^5^ activated T cells were sufficient to induce an abscess after 48 h (data not shown). T cells stimulated with wt WTA induced abscess formation in mice, whereas T cells stimulated with *dltA* WTA, naive T-cells, or APCs incubated with WTA in the absence of T cells were significantly less potent (Fig. 6B).

To lend further evidence to the premise that T cells are crucial for *S. aureus* abscess formation, we used CD4 T cell deficient mice. As predicted, WTA provoked abscesses in C57Bl/6 mice, but not in CD4-deficient C57Bl/6 tm1mac mice (Fig. 6C). These in vivo data clearly demonstrate the link between WTA dependent T cell activation and the modulation of abscess formation by activated CD4 T cells.

## Discussion

Ribitol-phosphate WTA is a conserved cell wall-associated ZPS produced by *S. aureus.* CP produced by the majority of staphylococcal strains may mask WTA on the bacterial surface. However, neither CP5 nor CP8 is expressed by 20–25% of clinical isolates [17], [18]. Even in encapsulated strains, WTA is most likely surface exposed during the logarithmic growth phase when little CP is produced [16], [17], [33]. WTA is present both in the logarithmic and stationary phases of bacterial growth, and it appears to be expressed constitutively by the bacterial cell. Structural genes encoding enzymes of WTA biosynthesis machinery can be upregulated under certain in vivo conditions, such as the early phases of nasal colonization, as demonstrated in the cotton rat model [34].

We were interested in the role of WTA in the development of skin infections caused by *S. aureus,* since these are among the most frequent staphylococcal infections. Moreover, WTA was shown to induce abscesses in a rat model of intraabdominal abscess formation [12]. Our data demonstrate that *S. aureus* WTA plays a critical role in the development of staphylococcal skin lesions. Mutants that lack WTA (*tagO*) or have an altered WTA structure (*dltA*) are significantly impaired in the mouse s.c. abscess model. The attenuated phenotype of the *dltA* mutant may be partially attributed to its susceptibility to killing by neutrophils and cationic microbial peptides [9], [13], which are highly abundant in skin infections [35]. The absence of *S. aureus* WTA or the presence of an altered WTA structure influences the molecular events that modulate abscess formation since purified wt WTA induced abscesses more efficiently than *dltA* WTA. T cells have previously been shown to play a role in animal models of *S. aureus* intraperitoneal and subcutaneous abscess formation [12], [22] and in a staphylococcal surgical wound infection model [22]. In this study we identify a novel role for zwitterionic WTA, together with CP, in the induction of subcutaneous abscess formation through a mechanism dependant upon T-cell activation. We demonstrated that purified WTA stimulated CD4+ T-cell proliferation in vitro, and that this was dependent upon the zwitterionic charge of WTA. WTA was unable to induce T cell proliferation when APCs lacking MHC II were used in co–culture, and the stimulatory activity of wt WTA, but not the residual activity of the *dltA* WTA, was diminished by HLA-DR blocking Abs. In contrast, inhibitors of intracellular processing pathways blocked the activity of wt WTA and the residual activity of *dltA* WTA. This argues for a charge-dependency of WTA presentation but not intracellular processing, which is consistent with reports characterizing other bacterial ZPS polymers [36]. Thus, WTA qualifies as a bacterial ZPS that is processed and presented by APCs via the MHCII pathway [19]. Having defined a mechanism by which WTA can activate T-cells, we then established that the T cell stimulatory activity of WTA could modulate skin abscess formation. CD4 T cells activated by purified wt WTA were sufficient to provoke abscesses when injected s.c. into the flanks of healthy mice. Moreover, CD4−/− mice injected with WTA failed to develop abscesses. Taken together, we present a novel function of WTA as a ZPS that can modulate CD4 T cell-dependent development of skin abscesses in mice.

*S. aureus* mutants defective in CP production were attenuated in the s.c. abscess model of infection, but purified CP8 was less active than WTA in T-cell proliferation assays and in the skin abscess model. Both CP8 and WTA showed similar potency in the rat intraperitoneal abscess model [12], suggesting that the underlying molecular mechanisms leading to abscess formation differ between the skin and peritoneal cavity. USA300 strains that are responsible for most SSTIs in the U.S. are negative for CP production [37], and thus WTA may be a critical factor that promotes USA300 abscess formation in humans.

The molecular effector mechanisms that are downstream of WTA-induced T cell activation and lead to abscess formation have yet to be resolved. CD4+ T cells have been shown to influence neutrophil recruitment into *S. aureus*–infected wound tissues via production of IFN-γ [26]. High local IFN-γ production leads to upregulation of CXC chemokines, thereby resulting in a pronounced neutrophil influx into the infected host tissues [26]. Here we provide evidence that *S. aureus* WTA and CP are both crucial for SSTIs provoked by *S. aureus,* and that WTA induces skin abscesses through MHC II–dependent activation of CD4+ T cells. A recent study by Cho et al. demonstrated the importance of γδ T cells in controlling neutrophil recruitment and influencing the outcome of intradermal *S. aureus* infections in mice [38]. The discrepancies between the results of Cho et al. and our own results may reflect subtle differences between the two infection models, as well as characteristics of the *S. aureus* strains utilized by the different labs. The immune mechanisms modulating the onset of infection likely differ depending on the site of infection, the specific virulence factors produced by the microbe, and the repertoire of effector cells responding to the bacterial insult.

Multiple virulence determinants likely affect *S. aureus* induction of SSTIs [39], and the host response to infection is currently under investigation by several laboratories. WTA is present in all *S. aureus* strains, and synergistic interactions among leukocidins, CPs, α-toxin [40], phenol-soluble modulins [41] and WTA may increase the frequency or severity of staphylococcal SSTIs. The ribitol phosphate WTA produced by *S. aureus* differs from the glycerol phosphate WTA polymer synthesized by *Staphylococcus epidermidis,* the most common staphylococcal species found on human skin [42], [43]. Whether the biological properties of the two polymers differ is a question that has not yet been addressed.

Our results demonstrate a novel function for the zwitterionic WTA polymer of *S. aureus*. We link the novel finding that WTA can stimulate CD4+ T cells to the development of staphylococcal SSTIs in an *in vivo* infection model. We offer new insights into the pathology of SSTIs caused by *S. aureus* and propose a novel host mechanism involved in staphylococcal skin infections.

## Methods

### Ethics Statement

Human blood was collected from healthy volunteers giving written informed consent for venipuncture, as approved by the Institutional Review Board of The Brigham and Women's Hospital (Human Subject Assurance Number 00000484). Animal experiments were performed in accordance with the guidelines of the Harvard Medical School Standing Committee on Animals (Animal Welfare Assurance Number A3431-01) under approved protocol 86-02131. The Harvard Medical School animal management program is accredited by the American Association for Accreditation of Laboratory Animal Care and meets National Institutes of Health standards as set forth in “Guide for the Care and Use of Laboratory Animals” (DHSS Publication No. (NIH) 85-23 Revised 1985). The institution also accepts as mandatory the Public Heath Service “Policy on Humane Care and Use of Laboratory Animals by Awardee Institutions” and NIH “Principles for the Utilization and Care of Vertebrate Animals Used in Testing, Research and Training.”

### Bacterial Strains and Growth Conditions

*S. aureus* SA113 (ATCC 35556) is a previously described laboratory strain [44]. The WTA-deficient Sa113 *tagO* mutant was generated by replacing the *tagO* gene with an erythromycin resistance cassette [9]. The Δ*dltA* mutant of *S. aureus* SA113 was generated by replacing the *dltA* gene with a spectinomycin resistance cassette [13], and this mutation was transduced into strain Newman with phage 80α. The phenotype of the *dltA* mutant was assessed by its ability to bind more positively charged cytochrome C (Sigma) than the wildtype strain, as described previously [45]. *S. aureus* Newman and the isogenic Newman *cap*5*O* mutant were described previously [46]. The *tagO::tet* mutation from RN4220 *tagO::tet geh::tagO* (kindly provided by Drs. Timothy Meredith and Suzanne Walker, Harvard Medical School) was introduced into strain Newman by transduction with phage 80α. The strain Newman *tagO* mutation was verified by PCR, and the absence of WTA was confirmed by measuring the phosphorus content of cell wall fractions prepared from the isogenic wild-type and mutant strains [9]. For genetic complementation studies, the mutants were complemented with a plasmid containing a wild-type copy of the *tagO* gene (pRB*tagO*) [9]. Bacterial strains were grown in tryptic soy broth (BBL) or B-Medium BM [9] unless otherwise noted.

### WTA Isolation and Purification

Staphylococcal WTA was isolated and analyzed as described [9]. The phosphorus content in WTA samples was determined by colorimetric assays [9], [13]. WTA was then dialyzed against 20 mM Bis/Tris buffer and run on a Q-Sepharose column with a NaCl gradient (0–1 M) followed by a Sephacryl S-300 size exclusion column. All buffers and water were pyrogen-free. The instruments and devices used in the WTA purification process were deproteinated by treatment with sulfuric acid and depyrogenated by heat inactivation for 4 h at 240°C or by treatment with a 1–2 M sodium hydroxide buffer. WTA was analyzed for protein by the Biorad method and by UV absorbance at 280 nm; for nucleic acid by UV absorbance at 260 nm and by agarose gel electrophoresis; and for endotoxin by the Limulus amebocyte lysate test (Charles River Laboratories, Charleston, SC). ^1^H-nuclear magnetic resonance spectroscopy (NMR) was performed for structural evaluation.

### Mouse Model of Subcutaneous Abscess Formation

Male Swiss Webster ND4 mice (4–6 weeks old) were purchased from Charles River or Harlan, and C57Bl/6 and C57Bl/6 tm1mac CD4−/− mice were purchased from The Jackson Laboratory. 10^4^ to 10^6^ CFU *S. aureus* or purified WTA was mixed with sterile dextran beads (Cytodex 1, Sigma), and the mixture (0.2 ml) was injected s.c. into the shaved flanks of mice as described previously [47]. The abscesses were either weighed or homogenized in TSB for quantitative culture. Alternatively, the myeloperoxidase (MPO) activity in abscess homogenates was measured with a colorimetric assay [48]. For histological analysis, excised tissue was fixed in formalin, embedded in paraffin, and stained with hematoxylin and eosin for microscopic examination.

### T-Cell Activation, Neutrophil Chemotaxis, and Cytokine Production Assays

Mononuclear cells were purified by density gradient centrifugation in Polymorphoprep (Axis-Shield) from blood drawn from various healthy human donors. Total T cells were purified from the mononuclear cell fraction with nylon wool columns (Polysciences, Inc.), and CD4 T cells were purified with CD4 enrichment columns (R&D Systems). Purity was assessed by FACS with CD3 and CD4 antibody staining. T cells (1×10^5^) were cultured with 1×10^5^ irradiated antigen-presenting cells and *S. aureus* WTA. Dose-dependency experiments were performed using WTA at concentrations of 20 and 2 µg/ml using SEA (5 ng/ml) and polygalacturonic acid (20 µg/ml) as positive and negative controls, respectively. Cellular proliferation was measured after 6 days by a ^3^H-thymidine incorporation method [21]. In some experiments splenic mouse T cells were purified over nylon wool columns and stimulated with WTA as described above. In addition, T-cell proliferation assays were performed in the presence of blocking Abs to the MHC class I molecules HLA-A, HLA-B, and HLA-C, the MHC class II molecules HLA-DR, HLA-DP, and HLA-DQ, and their respective isotype controls. The human Burkitt lymphoma cell line (Raji) expressing MHC class II and MHC class I molecules and its MHC class II transcriptional mutant cell line (RJ2.2.5) were used as APCs in certain experiments [21]. WTA was incubated with T cells purified from human blood and either MHCII+/+ Raji B-cells or MHCII−/− RJ 2.5.5 cells for 6 days, and cellular proliferation was measured as described above. WTA was tested for chemotactic activity on human neutrophils as previously described [49].

For cytokine detection assays HEK293 cells were cultured in Iscove's Modified Dulbecco's Medium (Invitrogen) with 10% fetal calf serum. THP-1 human monocytes were cultured in RPMI 1640 medium supplemented with 2 mM L-glutamine and 10% fetal calf serum. RAW 264.7 mouse macrophages were cultured in Dulbecco's modified Eagle's medium (GIBCO) with 10% fetal calf serum.

After stimulation with purified WTA, LPS, MALP-2 or Pam3Cys for 8 h (TNF alpha) or 10 h (IL-8), supernatants were harvested and stored at −20°C until assayed by ELISA (R&D Biosystems) according to the manufacturer's instructions. All assays were performed with triplicate or quadruplicate replicas.

### Adoptive Transfer Assays

The role of T-cell activation by WTA in the modulation of abscess formation was determined by transferring T cells stimulated with WTA into mice. Mouse T cells purified with nylon wool columns were cultured in vitro for 6 d with irradiated APCs and either 20 µg wt or *dltA* WTA. CD4+ T cells were isolated from the co-culture with mouse CD4+ enrichment columns (R&D Systems), mixed with cytodex beads, and 3×10^5^ T cells were injected s.c. into the flanks of mice. Control mice were injected with naïve T cells isolated from co-cultures with APCs and no stimulating antigen, APCs incubated with WTA (no T cells), or cytodex beads alone. Abscess formation was quantified by MPO activity.

## Supporting Information

Figure S1 Characterization of *tagO* and *dltA* mutants. A) WTA content of the wt and *tagO* mutant as determined by phosphorous content of cell wall fractions in Newman and C) the isogenic CP5 mutant *cap5O*. The *tagO* mutants show only residual Pi, which indicates the lack of WTA. Growth was monitored in LB broth after inoculation from a pre culture grown to log-phase (B and D). E) WTA content of the Newman wt and *dltA* mutant as determined by the phosphorous content of cell wall fractions. WT and mutant had similar amounts of WTA. F) Cytochrome C binding to whole cells. Bacterial cells in PBS were incubated with 0.5 µg/ml cytochrome C. The positive charge conferred by the ester linked D-alanine in the wt WTA diminishes binding of the positively charged cytochrome C. This observed phenotype of the *dltA* mutant depends on the lack of D-alanine esters.
(5.66 MB TIF)

Figure S2 Abscess formation with purified WTA. 20 µg of wt WTA or *dltA* WTA was mixed with cytodex beads and injected s.c into the flanks of mice. After 48 h, the mice were euthanized and representative abscesses photographed (A). The abscesses were excised, fixed in formalin, embedded in paraffin, and stained with hematoxylin and eosin for histological analysis (B).
(3.62 MB TIF)
