# Supplementary figures and images for: The Zwitterionic Cell Wall Teichoic Acid of Staphylococcus aureus Provokes Skin Abscesses in Mice by a Novel CD4+ T-Cell-Dependent Mechanism
Source: PLoS One. 2010 Oct 7;5(10):e13227. doi: 10.1371/journal.pone.0013227 (PMC2951347; doi:10.1371/journal.pone.0013227)

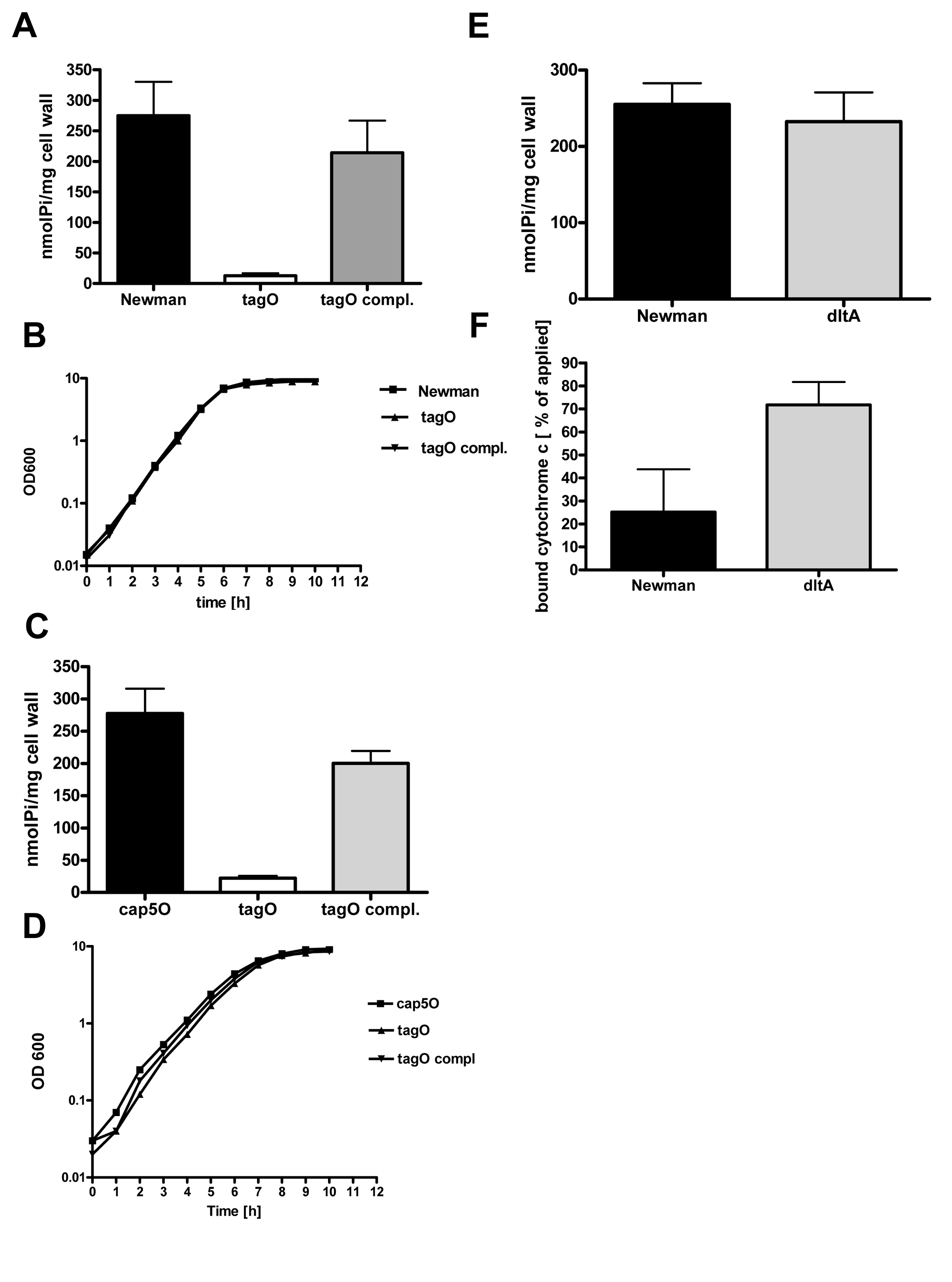

Supplement: Figure S1 — Characterization of tagO and dltA mutants. A) WTA content of the wt and tagO mutant as determined by phosphorous content of cell wall fractions in Newman and C) the isogenic CP5 mutant cap5O. The tagO mutants show only residual Pi, which indicates the lack of WTA. Growth was monitored in LB broth after inoculation from a pre culture grown to log-phase (B and D). E) WTA content of the Newman wt and dltA mutant as determined by the phosphorous content of cell wall fractions. WT and mutant had similar amounts of WTA. F) Cytochrome C binding to whole cells. Bacterial cells in PBS were incubated with 0.5 µg/ml cytochrome C. The positive charge conferred by the ester linked D-alanine in the wt WTA diminishes binding of the positively charged cytochrome C. This observed phenotype of the dltA mutant depends on the lack of D-alanine esters. (5.66 MB TIF) [file pone.0013227.s001.tif]

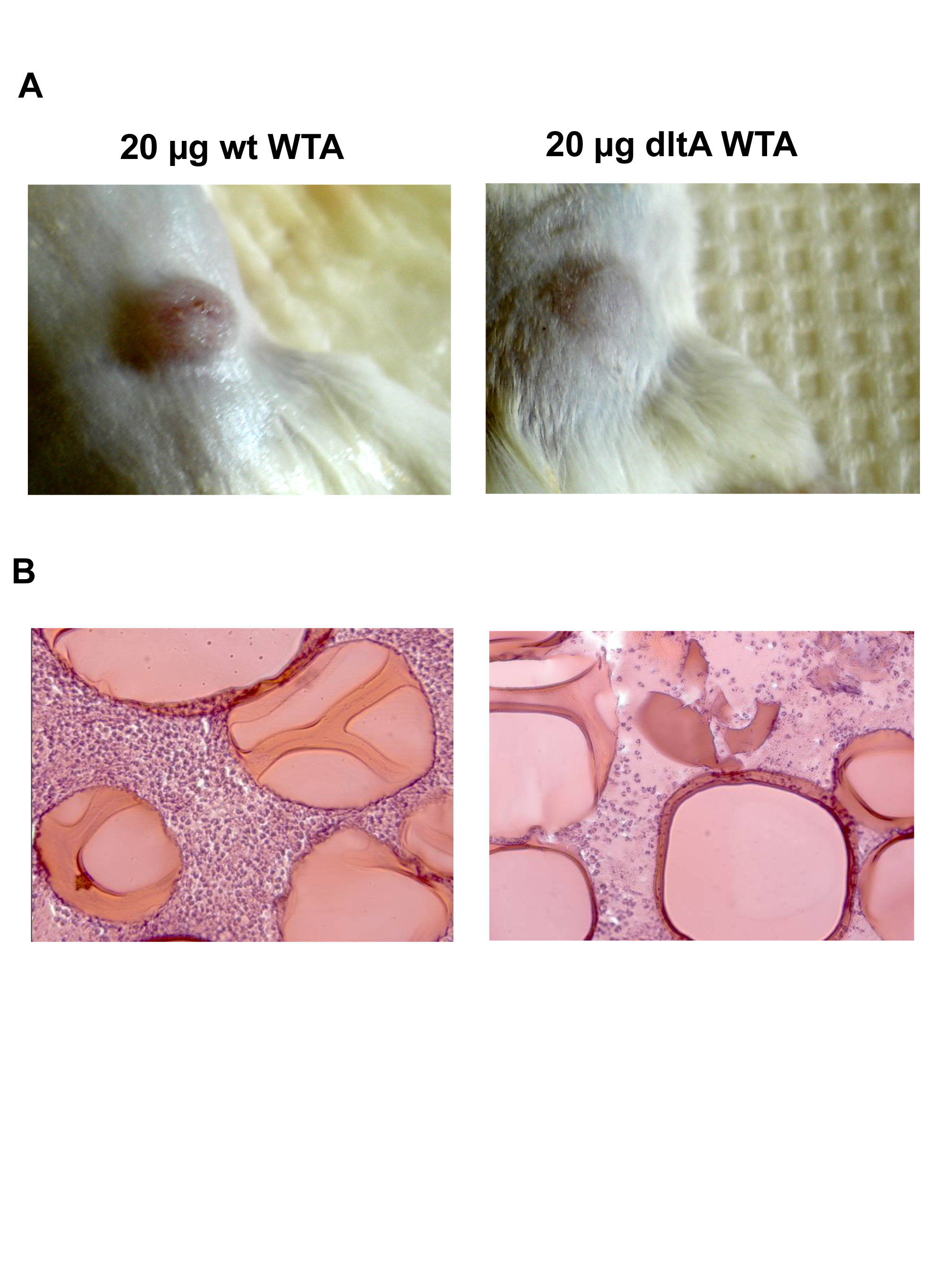

Supplement: Figure S2 — Abscess formation with purified WTA. 20 µg of wt WTA or dltA WTA was mixed with cytodex beads and injected s.c into the flanks of mice. After 48 h, the mice were euthanized and representative abscesses photographed (A). The abscesses were excised, fixed in formalin, embedded in paraffin, and stained with hematoxylin and eosin for histological analysis (B). (3.62 MB TIF) [file pone.0013227.s002.tif]
